# Family Involvement in the Care of Hospitalized Older Adults: Protocol for a Qualitative Evidence Synthesis

**DOI:** 10.2196/53255

**Published:** 2024-05-10

**Authors:** Judith B Vick, Blair P Golden, Sarah Cantrell, Melissa Louise Harris-Gersten, Mollie R Selmanoff, Susan Nicole Hastings, Tolu O Oyesanya, Karen M Goldstein, Courtney Van Houtven

**Affiliations:** 1 Durham VA Health Care System Durham, NC United States; 2 Department of Medicine Duke University School of Medicine Durham, NC United States; 3 National Clinician Scholars Program Duke Clinical and Translational Science Institute Durham, NC United States; 4 Department of Medicine University of Wisconsin School of Medicine and Public Health Madison, WI United States; 5 Medical Center Library and Archives Duke University School of Medicine Durham, NC United States; 6 Department of Care Management Johns Hopkins Hospital Baltimore, MD United States; 7 Duke University School of Nursing Durham, NC United States; 8 Department of Population Health Sciences Duke University School of Medicine Durham, NC United States

**Keywords:** systematic review, family, patient participation, hospital medicine, aged, geriatrics, qualitative research

## Abstract

**Background:**

Older adults are frequently hospitalized. Family involvement during these hospitalizations is incompletely characterized in the literature.

**Objective:**

This study aimed to better understand how families are involved in the care of hospitalized older adults and develop a conceptual model describing the phenomenon of family involvement in the care of hospitalized older adults.

**Methods:**

We describe the protocol of a qualitative evidence synthesis (QES), a systematic review of qualitative studies. We chose to focus on qualitative studies given the complexity and multifaceted nature of family involvement in care, a type of topic best understood through qualitative inquiry. The protocol describes our process of developing a research question and eligibility criteria for inclusion in our QES based on the SPIDER (Sample, Phenomenon of Interest, Design, Evaluation, and Research type) tool. It describes the development of our search strategy, which was used to search MEDLINE (via Ovid), Embase (via Elsevier), PsycINFO (via Ovid), and CINAHL Complete (via EBSCO). Title and abstract screening and full-text screening will occur sequentially. Purposive sampling may be used depending on the volume of studies identified as eligible for inclusion during our screening process. Descriptive data regarding included individual studies will be extracted and summarized in tables. The results from included studies will be synthesized using qualitative methods and used to develop a conceptual model. The conceptual model will be presented to community members via engagement panels for further refinement.

**Results:**

As of September 2023, we have assembled a multidisciplinary team including physicians, nurses, health services researchers, a librarian, a social worker, and a health economist. We have finalized our search strategy and executed the search, yielding 8862 total citations. We are currently screening titles and abstracts and anticipate that full-text screening, data extraction, quality appraisal, and synthesis will be completed by summer of 2024. Conceptual model development will then take place with community engagement panels. We anticipate submitting our manuscript for publication in the fall of 2024.

**Conclusions:**

This paper describes the protocol for a QES of family involvement in the care of hospitalized older adults. We will use identified themes to create a conceptual model to inform further intervention development and policy change.

**Trial Registration:**

PROSPERO 465617; https://www.crd.york.ac.uk/prospero/display_record.php?ID=CRD42023465617

**International Registered Report Identifier (IRRID):**

PRR1-10.2196/53255

## Introduction

Older adults comprise less than 20% of the US population [1], yet they account for over 40% of hospitalizations at 13.2 million per year [2]. These hospitalizations confer increased risks of infection, functional decline, and cognitive disability [3-7]. There is an urgent need to identify strategies to mitigate the negative sequelae associated with these hospitalizations both by preventing those that are preventable and attenuating the harmful effects of those that are not. Improving family involvement in care may be one strategy to mitigate negative downstream consequences of hospitalization for older adult patients [8-10]. For example, systematic family involvement in the discharge planning process reduces the risk of readmission for hospitalized older adults and is now written into policy in 42 states by the Care Act [11,12]. Implementation of the Care Act is associated with measures of improved patient experience, such as communication with nurses and physicians and receipt of discharge information [12]. There is a critical need to better understand family involvement in the full range of care for hospitalized older adults in addition to discharge planning.

Family involvement in older adults’ care has been better described in community and outpatient settings. Up to 36 million family members and friends provide care to community-dwelling older adults in the United States through medication management, care coordination, personal care, and nursing assistance [13]. This work totals roughly 30 billion hours of care annually [14]. A meta-analytical review showed that roughly 40% of adult patients are accompanied by family to primary care visits [15]. This review showed that the presence of family is associated with greater biomedical information giving while increasing the average visit length by 20%, findings that may contribute to patient-centered outcomes like satisfaction with care and that have consequences for workflow and reimbursement [15]. Other work has shown that families participate in ways that can be both helpful and harmful to communication, satisfaction, and the delivery of care in outpatient visits [15-19]. There was some national attention to the importance of family for the delivery of care for hospitalized patients (eg, in information exchange and decision-making) with the imposition of hospital visitor restrictions at the onset of the COVID-19 pandemic [20-22]. However, the full range of extant qualitative evidence describing the roles, tasks, experiences, and outcomes of family involvement in care for hospitalized older adults has not been systematically reviewed, and a conceptual model for this topic has not been developed in the peer-reviewed literature.

**Figure 1:**
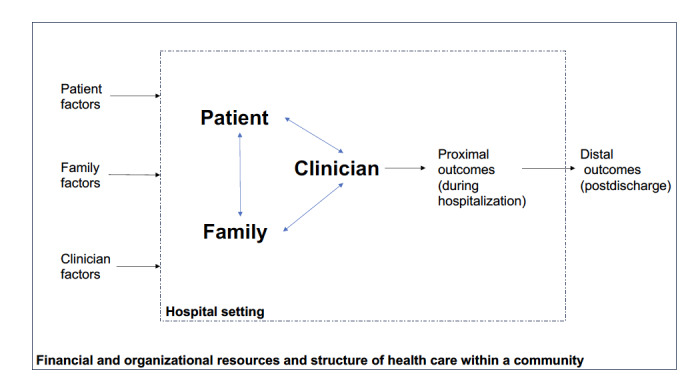
Preliminary conceptual model of family involvement in the care of hospitalized older adults (adapted from Wolff and Roter [15] with permission from Elsevier). Triadic relationships among patients, families, and clinicians in the hospital setting occur within the broader context of the financial and organizational resources and structure of health care within a community and are affected by prehospitalization patient factors, family factors, and clinician factors. The nature of the triadic involvement of patients, families, and clinicians in the hospital setting may affect proximal outcomes during a hospitalization and more distal outcomes post discharge.

Our objective in this study is to better understand how families are involved in the care of hospitalized older adults. An improved understanding of the interactions of family members and the professional medical team can inform future process innovation and policy change. To accomplish our objective, we chose to conduct a systematic review of qualitative studies, known as a qualitative evidence synthesis (QES), regarding family involvement in the care of hospitalized older adults. We chose to focus on qualitative research given that the complex nature of family involvement may be incompletely described through quantitative data. The themes identified in the review will be used to develop a conceptual model of family involvement in the care of hospitalized older adults, building on a prior model of family involvement in other settings (Figure 1) [15]. We anticipate that our refined model will establish a shared language for future multidisciplinary work, inform future intervention development and testing, and motivate workflow and policy changes [23]. This paper describes the protocol for our QES.

## Methods

### Overview

The research question of this review is as follows: How are families involved in the care of hospitalized older adults? Qualitative research provides in-depth understanding of human experience and multifaceted social phenomena that cannot be conveyed as richly with quantitative data alone [24]. Family involvement in the care of hospitalized older adults is this kind of complex social phenomenon. We chose a QES, a systematic review of qualitative evidence, for this study given our goal to be comprehensive in characterizing the existing literature on the complex phenomenon of family involvement in the care of hospitalized older adults [25]. We have submitted our review protocol on PROSPERO (ID 465617) and adhere to PRISMA-P (Preferred Items for Reporting Systematic Reviews and Meta-Analyses Protocols) guidelines in this protocol (Multimedia Appendix 1) [26,27]. The review results will be reported in the final paper using the ENTREQ (Enhancing Transparency in Reporting Synthesis of Qualitative Research) guidelines [24].

### Eligibility Criteria

Key elements of eligibility for inclusion in the review were specified using the SPIDER (Sample, Phenomenon of Interest, Design, Evaluation, Research type) tool, a standardized approach to systematic review eligibility criteria for qualitative systematic reviews as an alternative to the PICOT (Population, Intervention, Comparison, Outcome, Time) tool used for quantitative systematic reviews [28]. We define older adults as adults 65 years of age or older or as defined by an individual study’s authors as studying “older adults.” We use the term family broadly to refer to any family member, friend, or neighbor who is involved in a hospitalization of an older adult motivated by a personal relationship rather than financial remuneration [29]. For the purposes of eligibility in our QES, we do not distinguish between “family members” and “family caregivers” or require that this person be involved in the care of the older adult before or after a hospitalization. We define involvement broadly as inclusion in care processes and include search strategy cognate concepts such as engagement, participation, teamwork, collaboration, and coproduction [30,31]. Only original research studies using primary qualitative data collection and analysis methods and published in peer-reviewed journals are eligible for inclusion. Reference lists of reviews or protocols identified in our search will be manually checked to identify completed original studies that may be eligible in our review. Full inclusion and exclusion criteria, based on our SPIDER question, appear in Table 1.

**Table 1:** Sample, Phenomenon of Interest, Design, Evaluation, Research type criteria of eligibility for inclusion in our qualitative evidence synthesis.

Element and definition		Inclusion criteria	Exclusion criteria
**Sample**			
	Who is the sample or population of interest?	Family of hospitalized older adults: A study is eligible for inclusion if the majority (>50%) of included patients are older than 65 years if the average age of patients is older than 65 years, or if the study authors define the patient population of interest as being “older adults” (eg, if a study purports to examine hospitalized older adults which authors define in their study as being age 60 years or older) If age of patients is not specified in the abstract but is otherwise appropriate for inclusion, the title and abstract will advance to full text review	Hospitalized older adult patients without family involvement Majority or average of included patients are not older adults (as defined by individual study authors or older than 65 years)
**Phenomenon of interest**			
	What do you hope to understand?	Family involvement in the care of hospitalized older adults The term family broadly refers to any family member, friend, or neighbor who is involved in a hospitalization of an older adult motivated by a personal relationship rather than financial remuneration Family involvement in care is defined broadly as any type of participation, engagement, interaction with the professional medical team, support, or caregiving The settings of interest include the following: Emergency department prior to inpatient admission Medical ward Medical or cardiac intensive care unit Transitions of care will be included if they include care prior to hospital discharge. If exclusively concerning care after discharge, it will not be included	Involvement in the care of older adults in in nonemergency department, nonmedical, or nonmedical or cardiac intensive care unit settings, such as the following: Surgical ward Surgical intensive care unit Emergency department encounters not leading to hospital admission Outpatient encounters Hospital at home Trauma centers Long-term acute care Burn units Inpatient rehabilitation Urgent care visits Psychiatric units Inpatient hospice If patient and family involvement are not at all considered separately (eg, “patient and family engagement” is considered as a single entity rather than distinguishing between patient engagement and family engagement), the paper will be excluded
**Design**			
	What types of study methods are you interested in?	Qualitative designs: Phenomenological studies Ethnographies Grounded theory studies Case studies Narrative inquiries Discourse analyses Conversation analyses Qualitative data collection techniques: Interviews Participant observation Focus groups Review of documents or artifacts Open-ended surveys Qualitative data analysis techniques: Thematic analysis Framework analysis Content analysis Qualitative components of mixed methods studies will be retained in the review	Quantitative studies: Experimental study studies Quasi-experimental studies Descriptive quantitative studies Reviews (though will identify primary research papers from references for consideration of inclusion) Protocols (although will use protocols to search for completed studies that meet inclusion criteria) Nonresearch: Editorials Personal essays Comments Gray literature
**Evaluation**			
	What are the evaluation outcomes? (These may be subjective such as feelings, attitudes, opinions, etc)	All outcomes, including the following: Roles of family Tasks performed by family Experiences of family in care delivery Impacts of family on patient care Perceptions of family by professional staff	None
**Research type**			
	What type of research best suits your question? (qualitative, quantitative, and mixed methods)	Qualitative Mixed methods (qualitative aspect)	Quantitative studies
**Language**			
	Language of publication	Any	None Non-English publications will not be included in the analysis, but citations will be included in the appendix.
**Years**			
	Year of publication	Any	None
**Countries**			
	Country where research took place	Any	None
**Publication types**			
	Type of publication	Original research studies in a peer-reviewed journal	Non-peer reviewed journals Dissertations Conference abstract Gray literature Lay press Books Multimedia

### Information Sources

Our review uses the following databases: MEDLINE (via Ovid), Embase (via Elsevier), PsycINFO (via Ovid), and CINAHL Complete (via EBSCO).

### Search Strategies

The search strategies used a mix of database-specific subject headings and keywords searched in the title and abstract for the following concepts: family, informal caregivers, older adults, acute care, and the hospital setting. The search was developed and executed by an expert medical librarian, with input on keywords from other authors. The search strategy was peer-reviewed by another expert librarian using a modified PRESS (Peer Review of Electronic Search Strategies) checklist [32]. The search was executed in all 4 databases on August 23, 2023 and yielded 8862 citations in total. The full reproducible search strategies for all databases are included here as a Multimedia Appendix 2.

### Study Records

#### Data Management

Citations resulting from the database searches were uploaded to Covidence (Veritas Health Innovation) [33] and duplicates were removed.

#### Study Selection

We first piloted our title and abstract screening process by having 4 members of the study team (JBV, BPG, MRS, and MHG), each independently screen the first 50 titles and abstracts according to the inclusion and exclusion criteria specified in our SPIDER criteria; we discussed discrepancies as a group until we reached consensus. We are currently in the title and abstract screening stage in which each title and abstract will be screened in Covidence by 2 reviewers (JBV, BPG, MRS, and MHG) from the study team. When disagreements cannot be resolved by discussion between the 2 reviewers (JBV, BPG, MRS, and MHG), a third study team member will adjudicate. Full texts of papers included for the next stage of screening will then be retrieved and uploaded to Covidence. Each full-text paper will be read by 2 reviewers from the study team and assessed for eligibility based on the inclusion and exclusion criteria, with disagreement again resolved through discussion. If the 2 reviewers are unable to reach consensus, a third reviewer from the study team will adjudicate. If data from a single research study are presented and analyzed in multiple papers, we will retain each paper in our review and will denote when a single study is included multiple times. We will include a PRISMA flow diagram in our final paper, which will include reasons for exclusion of papers at the full-text stage [34]. If a study team member has been involved in one of the papers being considered for inclusion in the review, that team member will not be involved in decisions regarding its inclusion or quality (ie, will not be involved in screening its eligibility for inclusion, data extraction, or methodological assessment).

#### Data Collection Process

A data extraction tool will be created (using Microsoft Excel or similar software) and piloted for use with 2 full-text publications by 2 members (JBV, BPG, MRS, and MHG) of the study team. After piloting and subsequent modification of the data extraction tool, data will be extracted by 1 team member for each included publication, with a second member auditing the data extraction for accuracy.

### Data Extraction Items, Outcomes, and Prioritization

Data elements to extract will include characteristics such as journal, journal discipline, publication date, study location, sample size, research design, data collection techniques, data analysis techniques, study setting, funding sources, and theoretical or conceptual framework. This descriptive data will be entered into the data extraction tool. The results section of each included study will also be extracted. All qualitative results related to family involvement in care will be extracted; we do not prespecify or prioritize particular outcomes of interest.

### Sampling of Studies

Should the number of eligible studies exceed our ability to perform a high-quality synthesis, we will use purposive sampling to select a sample of studies for synthesis and analysis [35]. We expect to use purposive sampling if the number of eligible studies after full-text review exceeds 40. We will prioritize studies that (1) take place in the United States (the review authors’ country), (2) include older adult patients with cognitive impairment (given the greater need for family involvement for patients with impaired decision-making capacity) [36], and (3) are data rich [37]. Data richness will be determined using a previously published data richness scale to score studies on a data richness scale ranging from 1 to 5, with a score of 4 or higher being considered data rich [37].

### Assessing the Quality of Included Studies

Two independent reviewers will independently assess the methodological quality of the included studies using the QualSyst tool for qualitative studies [38], with the resolution of final quality assessments reached through discussion. Quality assessments will be entered into Excel along with data extraction. We will report the findings of our quality assessment for each study in a table in the final manuscript.

### Data Synthesis

Descriptive data about the studies (eg, journal name, journal discipline, and publication date) will be presented in a table and summarized narratively. We will analyze and synthesize the results from included studies using qualitative methods. Specifically, the results will be uploaded from into qualitative data analysis software such as Dedoose (SocioCultural Research Consultants, LLC), which we will use to facilitate our synthesis [39]. We will choose a synthesis method appropriate to the available pool of evidence, guided by published guidance on doing so, with the expectation that we will likely use thematic synthesis [40-42]. Thematic synthesis includes coding the text of each study’s results to develop descriptive themes, which are then used to generate analytical themes [41]. We anticipate using both inductive and deductive methods to identify themes. Deductively, we will consider domains identified in prior models of family involvement in outpatient and long-term care settings and in decision-making including caregiving context; facility context; family tasks, actions, and roles (eg, information exchange, emotional support, and relationship rapport); and patient and family outcomes [15,18,43]. We will meet as a team regularly to discuss review progress and iterate on emerging themes and will present a summary of our findings in a Summary of Qualitative Findings table.

### Conceptual Model Development

Once we have formulated a list of themes, we will use them to develop a conceptual model of family involvement in the care of hospitalized older adults to inform intervention development, measurement approaches, and policy needs. We will build on previously published conceptual models of family involvement in health care processes to refine our conceptual model for family involvement in care during hospitalizations of older adults [15,43]. As lead author, JBV will draft the initial model from themes identified in our synthesis of included papers. The study team will then meet iteratively to refine the model. Afterwards, we will obtain community input by eliciting feedback on our themes and conceptual model from family caregiver informants as a form of member checking [44,45]. We plan to perform this step through the Duke Clinical and Translational Science Institute Community Engaged Research Initiative and the Durham VA Veteran Research Engagement Panel to gather feedback and further refine themes and the conceptual model. We expect this step to take place in the spring or summer of 2024.

### Review Author Reflexivity

Our multidisciplinary review team includes members with expertise in hospital medicine, general internal medicine, geriatric medicine, nursing, information science, evidence synthesis, social work, and health services research. Throughout all stages of the review process and subsequent model development, we will each be cognizant of how our own backgrounds, views, and beliefs could influence our decisions regarding study eligibility decisions and the interpretation of the data. We will include discussions of potential bias in our regular team meetings. As a review team, we have a variety of backgrounds, which may help to overcome systematic bias in decision-making and analysis. Seven have clinical backgrounds (4 in medicine: JBV, BPG, KMG, SNH; 2 in nursing: MHG and TOO; 1 in clinical social work: MRS). Five are family caregiving researchers (JBV, BPG, TOO, CVH, and MHG) and 4 of us have formal training in qualitative methods (JBV, BPG, MHG, and TOO). One is a senior medical librarian (SC) and 1 is a health economist (CVH). Four have formal training in evidence synthesis (JBV, SC, KMG, and TOO). Six have been involved in the care of hospitalized older adults as family members (JBV, MHG, MRS, SNH, KMG, and CVH). We all work in academic settings. As lead author, JBV will keep an account of the review process and decisions made to document and reflect as the project progresses.

## Results

The study was submitted for registration in PROSPERO (ID 465617). As of September 2023, we have assembled a multidisciplinary team, developed the research question and inclusion and exclusion criteria, and finalized our search strategy. We executed the search for all 4 databases (MEDLINE, Embase, PsycINFO, and CINAHL Complete), yielding 8862 citations. We are currently screening titles and abstracts and anticipate that full-text screening, data extraction, quality appraisal, and synthesis will take place by January 2024. Conceptual model development will then take place, with initial drafting by the study team followed by community input via Community Engaged Research Initiative and Durham VA Veteran Research Engagement Panel in the spring of 2024. We anticipate submitting our review manuscript in the fall of 2024.

## Discussion

### Summary

The importance of family involvement in health care delivery has been increasingly recognized [9,10,15,18,29], but family roles, tasks, and interactions with the professional clinical team in the hospital have received less attention than in the outpatient and community settings. Systematic review of family involvement in discharge planning for hospitalized patients supported policy implementation of the Care Act in the majority of US states requiring systematic family involvement in discharge planning [11]. More comprehensive evidence synthesis of how families are involved with other aspects of care for hospitalized older adults has the potential to similarly impact intervention development and policy change that can positively affect public health. The proposed work aims to complete this synthesis via a systematic review of existing qualitative literature regarding family involvement in the care of hospitalized older adults, followed by the development of a conceptual model of family involvement in the care of hospitalized older adults.

### Strengths and Limitations

The strengths of the proposed study include its comprehensive approach and the focus on an incompletely understood phenomenon of interest. Systematic reviews of qualitative research are growing in use, but our QES method can still be considered an innovative strength [46]. The multidisciplinary team, including physicians, nurses, a librarian, a social worker, a health economist, and health services researchers with expertise in qualitative methods, evidence synthesis, and clinical care, is another strength of the proposed study. Limitations will likely stem from ambiguity in the phenomenon of interest, which may lead us to miss relevant studies that exist in the literature. This ambiguity surrounding the similar concepts of involvement, engagement, participation, teamwork, and other terms has been previously described [30,32]. We attempt to ameliorate this potential limitation by including as many cognate concepts in our search strategy as keywords based on prior work. Another limitation is intentional and that is the lack of inclusion of quantitative research. It is possible that prior experimental or observational studies that collected quantitative data only would give important information about the experiences of family involvement in the care of older adults that we will not be able to assess in a review of qualitative data alone. However, we maintain that a qualitative approach will identify richer data than that available with existing quantitative data. We anticipate that our included studies will identify roles that family members play. This will help identify narrower topics of interest within the larger phenomenon of family involvement, which will be better suited to a systematic review of quantitative studies in the future (eg, a systematic review of interventions designed to involve families for the purposes of delirium prevention for older adults).

### Conclusions

This paper describes the protocol for a QES of family involvement in the care of hospitalized older adults. Themes identified through this review will be used to develop a conceptual model of the phenomenon of interest. We anticipate that the review and conceptual model will be used in the future to improve the health and well-being of hospitalized older adults and their families through optimizing family involvement.

## Appendix

Multimedia Appendix 1 PRISMA-P (Preferred Reporting Items for Systematic review and Meta-Analysis Protocols) 2015 checklist: recommended items to address in a systematic review protocol.

Multimedia Appendix 2 Search strategies.

## Abbreviations

ENTREQ: Enhancing Transparency in Reporting synthesis of Qualitative Research
PICOT: Population, Intervention, Comparison, Outcome, Time
PRESS: Peer Review of Electronic Search Strategies
PRISMA-P: Preferred Items for Reporting Systematic Reviews and Meta-Analyses Protocols
QES: qualitative evidence synthesis
SPIDER: Sample, Phenomenon of Interest, Design, Evaluation, Research type

## Data Availability

The data sets generated and analyzed during this study are available from the corresponding author on reasonable request.
