# Supplementary material for: Family Involvement in the Care of Hospitalized Older Adults: Protocol for a Qualitative Evidence Synthesis
Source: JMIR Res Protoc. 2024 May 10;13:e53255. doi: 10.2196/53255 (PMC11127142; doi:10.2196/53255)
Supplement: Multimedia Appendix 2 [file resprot_v13i1e53255_app2.docx]

**Appendix – Search Strategies**

Librarian Searcher: Sarah Cantrell, MLIS; Duke University Medical Center Library & Archives, Duke University School of Medicine

Peer-review of search conducted by: Leila Ledbetter, MLIS; Duke University Medical Center Library & Archives, Duke University School of Medicine

**Database: MEDLINE (via Ovid)**
Search date: 8/23/2023
*Note: Ovid MEDLINE® ALL 1946 to August 22, 2023*

| **Search Set** | **Search Strategy** | **Results** |
| --- | --- | --- |
| #1  *Family* | family/ or exp friends/ or family relations/ or exp adult children/ or exp family characteristics or exp sibling relations/ or exp spouses/ or exp siblings/ or exp community support/ or exp family support/ or (family or families or relatives or spouse or spouses or spousal or partner or partners or husband or husbands or wife or wives or "adult child" or "adult children" or "adult daughter" or "adult daughters" or "adult son" or "adult sons" or sibling or siblings or sister or sisters or stepsister or stepsisters or brother or brothers or stepbrother or stepbrothers or aunt or aunts or uncle or uncles or cousin or cousins or friend or friends or neighbor or neighbors or neighbour or neighbours or "significant other" or "significant others" or "next of kin" or boyfriend or boyfriends or girlfriend or girlfriends).ti,ab. or ((medical or health or healthcare) adj2 (proxy or proxies or surrogate or surrogates or surrogacy)).ti,ab. | 1557783 |
| #2 *Informal Caregivers* | exp caregivers/ and (informal or informally or nonprofessional or non-professional or non-clinical or nonclinical or unpaid).ti,ab. | 5959 |
| #3  *Informal Caregivers* | ((informal or informally or nonprofessional or non-professional or non-clinical or nonclinical or unpaid) adj5 ("care giver" or "care givers" or caregiver or caregivers or caregiving or caretaker or caretakers or "care taker" or "care takers" or carer or carers or careworker or careworker or care workers or careworkers or "care worker" or "care workers" or "support person" or "support persons" or "support people")).ti,ab. or ("lay caregiver" or "lay caregivers" or "lay carer" or "lay carers").ti,ab. or ((patient or patients or hospital) adj3 (visitor or visitors or guardian or guardians or companion or companions)).ti,ab. | 9956 |
| #4 | 1 or 2 or 3 | 1564165 |
| #5  *Engagement* | exp stakeholder participation/ or exp communication/ or exp decision making, shared/ or exp professional-family relations/ or (adherence or adherent or attitude or attitudes or activation or collaborate or collaborates or collaborating or collaboration or collaborations or collaborative or collaboratively or communicate or communicated or communicates or communicating or communication or compliance or compliant or contribute or contributed or contributes or contribution or contributions or conversation or conversations or coproduc* or co-produc* or decision or decision-making or decisions or discuss or discussed or discusses or discussing or discussions or empower or empowered or empowerment or empowers or empowering or engage or engaged or engagement or engages or engaging or expectation or expectations or experience or experiences or facilitate or facilitates or facilitated or facilitating or facilitation or facilitations or include or includes or included or including or inclusion or inclusive or integrate or integrated or integrates or integrating or integration or involve or involved or involvement or involves or involving or "joint effort" or "joint efforts" or partnership or partnerships or participate or participates or participating or participation or perceive or perceives or perception or perceptions or presence or planning or role or roles or support or supported or supports or supporting or supportive or synergy or synergistic or synergetic or talk or talked or talking or talks or teamwork or "team work" or visitation or visitations or "working together").ti,ab. or (("professional-family" or "professional family" or "provider-family" or "provider family" or "physician-family" or "provider-family" or "provider family" or "clinician-family" or "clinician family" or "nurse family" or "nurse-family") adj3 (relation or relations or relationships or interaction or interactions or interacting or communication or communications or communicate or communicated or communicating or preference or preferences or attitude or attitudes or behavior or behaviors or behaviour or behaviours)).ti,ab. or ((family or families or patient or patients) adj2 (centered-care or "centered care" or focused-care or "focused care")).ti,ab. | 15594120 |
| #6  *Patient Age / Characteristics* | exp aged/ or exp frail elderly/ or exp health services for the aged/ or exp dementia/ or exp geriatrics/ or ("older adult" or "older adults" or  "frail adult" or "frail adults" or "old person" or "older person" or "old people" or "older people" or "old folk" or "old folks" or "older folk" or "older folks" or elder or elders or elderly or geriatric or geriatrics or geriatrician or geriatricians or gerontology or gerontologist or gerontologists or dementia or dementias or Alzheimer or alzheimers or Alzheimer?s or "lewy body" or "lewy bodies").ti,ab. or ((aged or aging or senior or seniors) adj2 (citizens or adult or adults or patient or patients or inpatient or inpatients or in-patient or in-patients or people or person or man or men or woman or women or population or populations)).ti,ab. | 3956495 |
| #7  *Setting: Acute care* | hospital units/ or intensive care units/ or exp coronary care units/ or ("critical care" or "intensive care" or icu or icus or "coronary care unit" or "coronary care units" or ccu or ccus).ti,ab. or ((hospital or hospitals) adj3 ("urgent care" or "emergency care")).ti,ab. or ((hospital or medical or medicine) adj2 (unit or units or ward or wards)).ti,ab. or ((admit or admits or admitted or admitting or admittance or admission or admissions) and ("acute care" or "acute illness" or "acute illnesses" or "acutely ill" or "emergency care" or "emergency service" or "emergency services" or "emergency department" or "emergency departments" or "emergency room" or "emergency rooms")).ti,ab. | 334553 |
| #8  *Setting:*  *hospitalization* | exp inpatients/ or exp hospitalization/ or (inpatient or inpatients or in-patient or in-patients or hospitalization or hospitalisation or hospitalizations or hospitalisations or "in-hospital" or inhospital).ti,ab. or ((hospitalized or hospitalised or admitted) adj3 (adult or adults or patient or patients or person or persons or people)).ti,ab. or (admitted adj2 (patient or patients)).ti,ab. | 2681062 |
| #9  Settings OR'd | 7 or 8 | 2867268 |
| #10  Combining of major concepts | 4 and 5 and 6 and 9 | 20971 |
| #11  *Remove Pediatrics only literature* | 10 not ((exp adolescent/ OR exp child/ OR exp infant/) NOT exp adult/) | 20753 |
| #12  *Qualitative designs* | exp Qualitative Research/ or exp Focus Groups/ or exp Interviews as Topic/ or exp Narration/ or exp personal narratives as topic/ or (qualitative or qualitatively or "focus group" or "focus groups" or "mixed method" or "mixed methods" or "thematic analysis" or "thematic analyses" or "content analysis" or "content analyses" or "key informant" or "key informants" or fieldwork or "field work" or "grounded theory" or phenomenology or phenomenological or "lived experience" or "lived experiences" or ethnograph or ethnographic or ethnography or ethnographies or autoethnography or autoethnographies or autoethnographic or "oral history" or "oral histories").ti,ab. or ((semi-structured or semistructured or unstructured or informal or in-depth or indepth or "face to face" or face-to-face or structured or guide or guides) and (interview* or discussion* or questionnaire*)).ti,ab. | 672495 |
| #13 | 11 and 12 | 3062 |
| #14 | 13 not (congress).pt. | 3062 |
| Exemplar validation check | 14 and (15991107 OR 16837693 OR 10686568 OR 36196458 OR 34923742).ui. | 5/5 |

**Database: Embase (via Elsevier)**
Search date: 8/23/2023
*Note: Search from the Results page*

| **Search Set** | **Search Strategy** | **Results** |
| --- | --- | --- |
| #1  *Family* | 'family'/de OR 'adult child'/de OR 'aunt'/exp OR 'cousin'/exp OR 'extended family'/exp OR 'family decision making'/exp OR 'family health'/exp OR 'family life'/de OR 'family functioning'/exp OR 'family interaction'/exp OR 'family relation'/de OR 'family support'/de OR 'sibling relation'/exp OR 'grandparent'/exp OR 'great-grandparent'/exp OR 'spouse'/exp OR 'uncle'/exp OR 'sibling'/exp OR 'friend'/exp OR 'community support'/exp OR (family OR families OR relatives OR spouse OR spouses OR spousal OR partner OR partners OR husband OR husbands OR wife OR wives OR 'adult child' OR 'adult children' OR 'adult daughter' OR 'adult daughters' OR 'adult son' OR 'adult sons' OR sibling OR siblings OR sister OR sisters OR stepsister OR stepsisters OR brother OR brothers OR stepbrother OR stepbrothers OR aunt OR aunts OR uncle OR uncles OR cousin OR cousins OR friend OR friends OR neighbor OR neighbors OR neighbour OR neighbours OR 'significant other' OR 'significant others' OR 'next of kin' OR boyfriend OR boyfriends OR girlfriend OR girlfriends):ti,ab OR ((medical OR health OR healthcare) NEAR/2 (proxy OR proxies OR surrogate OR surrogates OR surrogacy)):ti,ab | 1970134 |
| #2 *Informal Caregivers* | 'caregiver'/exp AND (informal OR informally OR nonprofessional OR non-professional OR non-clinical OR nonclinical OR unpaid):ti,ab | 8287 |
| #3  *Informal Caregivers* | ((informal OR informally OR nonprofessional OR 'non professional' OR 'non clinical' OR nonclinical OR unpaid) NEAR/5 ('care giver' OR 'care givers' OR caregiver OR caregivers OR caregiving OR caretaker OR caretakers OR 'care taker' OR 'care takers' OR carer OR carers OR careworker OR careworkers OR 'care worker' OR 'care workers' OR 'support person' OR 'support persons' OR 'support people')):ti,ab OR ('lay caregiver' OR 'lay caregivers' OR 'lay carer' OR 'lay carers'):ti,ab OR ((patient OR patients OR hospital) NEAR/3 (visitor OR visitors OR guardian OR guardians OR companion OR companions)):ti,ab | 13391 |
| #4 | #1 OR #2 OR #3 | 1978853 |
| #5  *Engagement* | 'stakeholder engagement'/exp OR 'interpersonal communication'/exp OR 'shared decision making'/exp OR 'collaborative care team'/exp OR (adherence OR adherent OR attitude OR attitudes OR activation OR collaborate OR collaborates OR collaborating OR collaboration OR collaborations OR collaborative OR collaboratively OR communicate OR communicated OR communicates OR communicating OR communication OR compliance OR compliant OR contribute OR contributed OR contributes OR contribution OR contributions OR conversation OR conversations OR coproduc* OR co-produc* OR decision OR decision-making OR decisions OR discuss OR discussed OR discusses OR discussing OR discussions OR empower OR empowered OR empowerment OR empowers OR empowering OR engage OR engaged OR engagement OR engages OR engaging OR expectation OR expectations OR experience OR experiences OR facilitate OR facilitates OR facilitated OR facilitating OR facilitation OR facilitations OR include OR includes OR included OR including OR inclusion OR inclusive OR integrate OR integrated OR integrates OR integrating OR integration OR involve OR involved OR involvement OR involves OR involving OR 'joint effort' OR 'joint efforts' OR partnership OR partnerships OR participate OR participates OR participating OR participation OR perceive OR perceives OR perception OR perceptions OR presence OR planning OR role OR roles OR support OR supported OR supports OR supporting OR supportive OR synergy OR synergistic OR synergetic OR talk OR talked OR talking OR talks OR teamwork OR 'team work' OR visitation OR visitations OR 'working together'):ti,ab OR (('professional-family' OR 'professional family' OR 'provider-family' OR 'provider family' OR 'physician-family' OR 'provider-family' OR 'provider family' OR 'clinician-family' OR 'clinician family' OR 'nurse family' OR 'nurse-family') NEAR/3 (relation OR relations OR relationships OR interaction OR interactions OR interacting OR communication OR communications OR communicate OR communicated OR communicating OR preference OR preferences OR attitude OR attitudes OR behavior OR behaviors OR behaviour OR behaviours)):ti,ab OR ((family OR families OR patient OR patients) NEAR/2 (centered-care OR 'centered care' OR focused-care OR 'focused care')):ti,ab | 20578469 |
| #6  *Patient Age / Characteristics* | 'aged'/de OR 'aged hospital patient'/de OR 'frail elderly'/de OR 'very elderly'/de OR 'elderly care'/de OR 'geriatric care'/exp OR 'geriatrics'/de OR 'dementia'/de OR 'Alzheimer disease'/de OR 'senile dementia'/exp OR ('older adult' OR 'older adults' OR 'frail adult' OR 'frail adults' OR 'old person' OR 'older person' OR 'old people' OR 'older people' OR 'old folk' OR 'old folks' OR 'older folk' OR 'older folks' OR elder OR elders OR elderly OR geriatric OR geriatrics OR geriatrician OR geriatricians OR gerontology OR gerontologist OR gerontologists OR dementia OR dementias OR Alzheimer OR alzheimers OR Alzheimer?s OR 'lewy body' OR 'lewy bodies'):ti,ab OR ((aged OR aging OR senior OR seniors) NEAR/2 (citizens OR adult OR adults OR patient OR patients OR inpatient OR inpatients OR in-patient OR in-patients OR people OR person OR man OR men OR woman OR women OR population OR populations)):ti,ab | 4504136 |
| #7  *Setting: Acute care* | 'hospital subdivisions and components'/de OR 'hospital bed'/de OR 'hospital department'/de OR 'ward'/de OR 'geriatric ward'/de OR 'high dependency unit'/de OR 'intensive care unit'/de OR 'coronary care unit'/de OR 'medical intensive care unit'/de OR 'oncology ward'/de OR 'psychiatric department'/de OR ('critical care' OR 'intensive care' OR icu OR icus OR 'coronary care unit' OR 'coronary care units' OR ccu OR ccus):ti,ab OR ((hospital OR hospitals) NEAR/3 ('urgent care' OR 'emergency care')):ti,ab OR ((hospital OR medical OR medicine) NEAR/2 (unit OR units OR ward OR wards)):ti,ab OR ((admit OR admits OR admitted OR admitting OR admittance OR admission OR admissions) AND ('acute care' OR 'acute illness' OR 'acute illnesses' OR 'acutely ill' OR 'emergency care' OR 'emergency service' OR 'emergency services' OR 'emergency department' OR 'emergency departments' OR 'emergency room' OR 'emergency rooms')):ti,ab | 650791 |
| #8  *Setting:*  *hospitalization* | 'aged hospital patient'/de OR 'hospital patient'/de OR 'hospitalization'/exp OR (inpatient OR inpatients OR in-patient OR in-patients OR hospitalization OR hospitalisation OR hospitalizations OR hospitalisations OR 'in-hospital' OR inhospital):ti,ab OR ((hospitalized OR hospitalised OR admitted) NEAR/3 (adult OR adults OR patient OR patients OR person OR persons OR people)):ti,ab OR (admitted NEAR/2 (patient OR patients)):ti,ab | 4276853 |
| #9  Settings OR'd | #7 OR #8 | 4646832 |
| #10  Combining of major concepts | #4 AND #5 AND #6 AND #9 | 30290 |
| #11  *Remove Pediatrics only literature* | #10 NOT ((adolescent/exp OR child/exp OR infant/exp) NOT adult/exp) | 29778 |
| #12  *Qualitative designs* | 'qualitative research'/exp OR 'focus group'/exp OR 'interview'/exp OR (qualitative OR qualitatively OR 'focus group' OR 'focus groups' OR 'mixed method' OR 'mixed methods' OR 'thematic analysis' OR 'thematic analyses' OR 'content analysis' OR 'content analyses' OR 'key informant' OR 'key informants' OR fieldwork OR 'field work' OR 'grounded theory' OR phenomenology OR phenomenological OR 'lived experience' OR 'lived experiences' OR ethnograph OR ethnographic OR ethnography OR ethnographies OR autoethnography OR autoethnographies OR autoethnographic OR 'oral history' OR 'oral histories'):ti,ab OR ((semi-structured OR semistructured OR unstructured OR informal OR in-depth OR indepth OR 'face to face' OR face-to-face OR structured OR guide OR guides) NEAR/6 (interview* OR discussion* OR questionnaire*)):ti,ab | 943345 |
| #13 | #11 AND #12 | 4124 |
| #14 | #13 NOT ([conference abstract]/lim OR 'conference abstract'/exp OR 'conference abstract'/it OR 'book'/de) | 3160 |

**Database: CINAHL Complete (via EBSCO)**
Search date: 8/23/2023

| **Search Set** | **Search Strategy** | **Results** |
| --- | --- | --- |
| #1  *Family* | (MH "Family") OR (MH "Adult Children") OR (MH "Extended Family") OR (MH "Grandparents") OR (MH "Nuclear Family") OR (MH "Family Structure") OR (MH "Spouses") OR (MH "Siblings") OR (MH "Sibling Relations") OR (MH "Family Support") OR ((TI family OR AB family) OR (TI families OR AB families) OR (TI relatives OR AB relatives) OR (TI spouse OR AB spouse) OR (TI spouses OR AB spouses) OR (TI spousal OR AB spousal) OR (TI partner OR AB partner) OR (TI partners OR AB partners) OR (TI husband OR AB husband) OR (TI husbands OR AB husbands) OR (TI wife OR AB wife) OR (TI wives OR AB wives) OR (TI "adult child" OR AB "adult child") OR (TI "adult children" OR AB "adult children") OR (TI "adult daughter" OR AB "adult daughter") OR (TI "adult daughters" OR AB "adult daughters") OR (TI "adult son" OR AB "adult son") OR (TI "adult sons" OR AB "adult sons") OR (TI sibling OR AB sibling) OR (TI siblings OR AB siblings) OR (TI sister OR AB sister) OR (TI sisters OR AB sisters) OR (TI stepsister OR AB stepsister) OR (TI stepsisters OR AB stepsisters) OR (TI brother OR AB brother) OR (TI brothers OR AB brothers) OR (TI stepbrother OR AB stepbrother) OR (TI stepbrothers OR AB stepbrothers) OR (TI aunt OR AB aunt) OR (TI aunts OR AB aunts) OR (TI uncle OR AB uncle) OR (TI uncles OR AB uncles) OR (TI cousin OR AB cousin) OR (TI cousins OR AB cousins) OR (TI friend OR AB friend) OR (TI friends OR AB friends) OR (TI neighbor OR AB neighbor) OR (TI neighbors OR AB neighbors) OR (TI neighbour OR AB neighbour) OR (TI neighbours OR AB neighbours) OR (TI "significant other" OR AB "significant other") OR (TI "significant others" OR AB "significant others") OR (TI "next of kin" OR AB "next of kin") OR (TI boyfriend OR AB boyfriend) OR (TI boyfriends OR AB boyfriends) OR (TI girlfriend OR AB girlfriend) OR (TI girlfriends OR AB girlfriends)) OR (((TI medical OR AB medical) OR (TI health OR AB health) OR (TI healthcare OR AB healthcare)) N2 ((TI proxy OR AB proxy) OR (TI proxies OR AB proxies) OR (TI surrogate OR AB surrogate) OR (TI surrogates OR AB surrogates) OR (TI surrogacy OR AB surrogacy))) | 756891 |
| #2 *Informal Caregivers* | (MH "Caregivers") OR ((TI informal OR AB informal) OR (TI informally OR AB informally) OR (TI nonprofessional OR AB nonprofessional) OR (TI non-professional OR AB non-professional) OR (TI non-clinical OR AB non-clinical) OR (TI nonclinical OR AB nonclinical) OR (TI unpaid OR AB unpaid)) | 49128 |
| #3  *Informal Caregivers* | (((TI informal OR AB informal) OR (TI informally OR AB informally) OR (TI nonprofessional OR AB nonprofessional) OR (TI non-professional OR AB non-professional) OR (TI non-clinical OR AB non-clinical) OR (TI nonclinical OR AB nonclinical) OR (TI unpaid OR AB unpaid)) N5 ((TI "care giver" OR AB "care giver") OR (TI "care givers" OR AB "care givers") OR (TI caregiver OR AB caregiver) OR (TI caregivers OR AB caregivers) OR (TI caregiving OR AB caregiving) OR (TI caretaker OR AB caretaker) OR (TI caretakers OR AB caretakers) OR (TI "care taker" OR AB "care taker") OR (TI "care takers" OR AB "care takers") OR (TI carer OR AB carer) OR (TI carers OR AB carers) OR (TI careworker OR AB careworker) OR (TI careworker OR AB careworker) OR (TI "care workers" OR AB "care workers") OR (TI careworkers OR AB careworkers) OR (TI "care worker" OR AB "care worker") OR (TI "care workers" OR AB "care workers") OR (TI "support person" OR AB "support person") OR (TI "support persons" OR AB "support persons") OR (TI "support people" OR AB "support people"))) OR ((TI "lay caregiver" OR AB "lay caregiver") OR (TI "lay caregivers" OR AB "lay caregivers") OR (TI "lay carer" OR AB "lay carer") OR (TI "lay carers" OR AB "lay carers")) OR (((TI patient OR AB patient) OR (TI patients OR AB patients) OR (TI hospital OR AB hospital)) N3 ((TI visitor OR AB visitor) OR (TI visitors OR AB visitors) OR (TI guardian OR AB guardian) OR (TI guardians OR AB guardians) OR (TI companion OR AB companion) OR (TI companions OR AB companions))) | 5234 |
| #4 | S1 OR S2 OR S3 | 794657 |
| #5  *Engagement* | (MH "Stakeholder Participation") OR (MH "Communication+") OR (MH "Decision Making, Shared") OR (MH "Professional-Family Relations") OR ((TI adherence OR AB adherence) OR (TI adherent OR AB adherent) OR (TI attitude OR AB attitude) OR (TI attitudes OR AB attitudes) OR (TI activation OR AB activation) OR (TI collaborate OR AB collaborate) OR (TI collaborates OR AB collaborates) OR (TI collaborating OR AB collaborating) OR (TI collaboration OR AB collaboration) OR (TI collaborations OR AB collaborations) OR (TI collaborative OR AB collaborative) OR (TI collaboratively OR AB collaboratively) OR (TI communicate OR AB communicate) OR (TI communicated OR AB communicated) OR (TI communicates OR AB communicates) OR (TI communicating OR AB communicating) OR (TI communication OR AB communication) OR (TI compliance OR AB compliance) OR (TI compliant OR AB compliant) OR (TI contribute OR AB contribute) OR (TI contributed OR AB contributed) OR (TI contributes OR AB contributes) OR (TI contribution OR AB contribution) OR (TI contributions OR AB contributions) OR (TI conversation OR AB conversation) OR (TI conversations OR AB conversations) OR (TI coproduc* OR AB coproduc*) OR (TI co-produc* OR AB co-produc*) OR (TI decision OR AB decision) OR (TI decision-making OR AB decision-making) OR (TI decisions OR AB decisions) OR (TI discuss OR AB discuss) OR (TI discussed OR AB discussed) OR (TI discusses OR AB discusses) OR (TI discussing OR AB discussing) OR (TI discussions OR AB discussions) OR (TI empower OR AB empower) OR (TI empowered OR AB empowered) OR (TI empowerment OR AB empowerment) OR (TI empowers OR AB empowers) OR (TI empowering OR AB empowering) OR (TI engage OR AB engage) OR (TI engaged OR AB engaged) OR (TI engagement OR AB engagement) OR (TI engages OR AB engages) OR (TI engaging OR AB engaging) OR (TI expectation OR AB expectation) OR (TI expectations OR AB expectations) OR (TI experience OR AB experience) OR (TI experiences OR AB experiences) OR (TI facilitate OR AB facilitate) OR (TI facilitates OR AB facilitates) OR (TI facilitated OR AB facilitated) OR (TI facilitating OR AB facilitating) OR (TI facilitation OR AB facilitation) OR (TI facilitations OR AB facilitations) OR (TI include OR AB include) OR (TI includes OR AB includes) OR (TI included OR AB included) OR (TI including OR AB including) OR (TI inclusion OR AB inclusion) OR (TI inclusive OR AB inclusive) OR (TI integrate OR AB integrate) OR (TI integrated OR AB integrated) OR (TI integrates OR AB integrates) OR (TI integrating OR AB integrating) OR (TI integration OR AB integration) OR (TI involve OR AB involve) OR (TI involved OR AB involved) OR (TI involvement OR AB involvement) OR (TI involves OR AB involves) OR (TI involving OR AB involving) OR (TI "joint effort" OR AB "joint effort") OR (TI "joint efforts" OR AB "joint efforts") OR (TI partnership OR AB partnership) OR (TI partnerships OR AB partnerships) OR (TI participate OR AB participate) OR (TI participates OR AB participates) OR (TI participating OR AB participating) OR (TI participation OR AB participation) OR (TI perceive OR AB perceive) OR (TI perceives OR AB perceives) OR (TI perception OR AB perception) OR (TI perceptions OR AB perceptions) OR (TI presence OR AB presence) OR (TI planning OR AB planning) OR (TI role OR AB role) OR (TI roles OR AB roles) OR (TI support OR AB support) OR (TI supported OR AB supported) OR (TI supports OR AB supports) OR (TI supporting OR AB supporting) OR (TI supportive OR AB supportive) OR (TI synergy OR AB synergy) OR (TI synergistic OR AB synergistic) OR (TI synergetic OR AB synergetic) OR (TI talk OR AB talk) OR (TI talked OR AB talked) OR (TI talking OR AB talking) OR (TI talks OR AB talks) OR (TI teamwork OR AB teamwork) OR (TI "team work" OR AB "team work") OR (TI visitation OR AB visitation) OR (TI visitations OR AB visitations) OR (TI "working together" OR AB "working together")) OR (((TI professional-family OR AB professional-family) OR (TI "professional family" OR AB "professional family") OR (TI provider-family OR AB provider-family) OR (TI "provider family" OR AB "provider family") OR (TI physician-family OR AB physician-family) OR (TI provider-family OR AB provider-family) OR (TI "provider family" OR AB "provider family") OR (TI clinician-family OR AB clinician-family) OR (TI "clinician family" OR AB "clinician family") OR (TI "nurse family" OR AB "nurse family") OR (TI nurse-family OR AB nurse-family)) N3 ((TI relation OR AB relation) OR (TI relations OR AB relations) OR (TI relationships OR AB relationships) OR (TI interaction OR AB interaction) OR (TI interactions OR AB interactions) OR (TI interacting OR AB interacting) OR (TI communication OR AB communication) OR (TI communications OR AB communications) OR (TI communicate OR AB communicate) OR (TI communicated OR AB communicated) OR (TI communicating OR AB communicating) OR (TI preference OR AB preference) OR (TI preferences OR AB preferences) OR (TI attitude OR AB attitude) OR (TI attitudes OR AB attitudes) OR (TI behavior OR AB behavior) OR (TI behaviors OR AB behaviors) OR (TI behaviour OR AB behaviour) OR (TI behaviours OR AB behaviours))) OR (((TI family OR AB family) OR (TI families OR AB families) OR (TI patient OR AB patient) OR (TI patients OR AB patients)) N2 ((TI centered-care OR AB centered-care) OR (TI "centered care" OR AB "centered care") OR (TI focused-care OR AB focused-care) OR (TI "focused care" OR AB "focused care"))) | 3847206 |
| #6  *Patient Age / Characteristics* | (MH "Aged+") OR (MH "Frail Elderly") OR (MH "Health Services for Older Persons") OR (MH "Dementia+") OR (MH "Geriatrics+") OR ((TI "older adult" OR AB "older adult") OR (TI "older adults" OR AB "older adults") OR (TI "frail adult" OR AB "frail adult") OR (TI "frail adults" OR AB "frail adults") OR (TI "old person" OR AB "old person") OR (TI "older person" OR AB "older person") OR (TI "old people" OR AB "old people") OR (TI "older people" OR AB "older people") OR (TI "old folk" OR AB "old folk") OR (TI "old folks" OR AB "old folks") OR (TI "older folk" OR AB "older folk") OR (TI "older folks" OR AB "older folks") OR (TI elder OR AB elder) OR (TI elders OR AB elders) OR (TI elderly OR AB elderly) OR (TI geriatric OR AB geriatric) OR (TI geriatrics OR AB geriatrics) OR (TI geriatrician OR AB geriatrician) OR (TI geriatricians OR AB geriatricians) OR (TI gerontology OR AB gerontology) OR (TI gerontologist OR AB gerontologist) OR (TI gerontologists OR AB gerontologists) OR (TI dementia OR AB dementia) OR (TI dementias OR AB dementias) OR (TI Alzheimer OR AB Alzheimer) OR (TI alzheimers OR AB alzheimers) OR (TI Alzheimer#s OR AB Alzheimer#s) OR (TI "lewy body" OR AB "lewy body") OR (TI "lewy bodies" OR AB "lewy bodies")) OR (((TI aged OR AB aged) OR (TI aging OR AB aging) OR (TI senior OR AB senior) OR (TI seniors OR AB seniors)) N2 ((TI citizens OR AB citizens) OR (TI adult OR AB adult) OR (TI adults OR AB adults) OR (TI patient OR AB patient) OR (TI patients OR AB patients) OR (TI inpatient OR AB inpatient) OR (TI inpatients OR AB inpatients) OR (TI in-patient OR AB in-patient) OR (TI in-patients OR AB in-patients) OR (TI people OR AB people) OR (TI person OR AB person) OR (TI man OR AB man) OR (TI men OR AB men) OR (TI woman OR AB woman) OR (TI women OR AB women) OR (TI population OR AB population) OR (TI populations OR AB populations))) | 317416 |
| #7  *Setting: Acute care* | ((MH "Hospital Units+") OR (MH "Intensive Care Units") OR (MH "Coronary Care Units") OR (MH "Oncology Care Units") OR (MH "Psychiatric Units")  OR ((TI "critical care" OR AB "critical care") OR (TI "intensive care" OR AB "intensive care") OR (TI icu OR AB icu) OR (TI icus OR AB icus) OR (TI "coronary care unit" OR AB "coronary care unit") OR (TI "coronary care units" OR AB "coronary care units") OR (TI ccu OR AB ccu) OR (TI ccus OR AB ccus)) OR (((TI hospital OR AB hospital) OR (TI hospitals OR AB hospitals)) N3 ((TI "urgent care" OR AB "urgent care") OR (TI "emergency care" OR AB "emergency care"))) OR (((TI hospital OR AB hospital) OR (TI medical OR AB medical) OR (TI medicine OR AB medicine)) N2 ((TI unit OR AB unit) OR (TI units OR AB units) OR (TI ward OR AB ward) OR (TI wards OR AB wards))) OR (((TI admit OR AB admit) OR (TI admits OR AB admits) OR (TI admitted OR AB admitted) OR (TI admitting OR AB admitting) OR (TI admittance OR AB admittance) OR (TI admission OR AB admission) OR (TI admissions OR AB admissions)) AND ((TI "acute care" OR AB "acute care") OR (TI "acute illness" OR AB "acute illness") OR (TI "acute illnesses" OR AB "acute illnesses") OR (TI "acutely ill" OR AB "acutely ill") OR (TI "emergency care" OR AB "emergency care") OR (TI "emergency service" OR AB "emergency service") OR (TI "emergency services" OR AB "emergency services") OR (TI "emergency department" OR AB "emergency department") OR (TI "emergency departments" OR AB "emergency departments") OR (TI "emergency room" OR AB "emergency room") OR (TI "emergency rooms" OR AB "emergency rooms"))) | 23372 |
| #8  *Setting:*  *hospitalization* | (MH "Inpatients") OR (MH "Hospitalization+") OR ((TI inpatient OR AB inpatient) OR (TI inpatients OR AB inpatients) OR (TI in-patient OR AB in-patient) OR (TI in-patients OR AB in-patients) OR (TI hospitalization OR AB hospitalization) OR (TI hospitalisation OR AB hospitalisation) OR (TI hospitalizations OR AB hospitalizations) OR (TI hospitalisations OR AB hospitalisations) OR (TI in-hospital OR AB in-hospital) OR (TI inhospital OR AB inhospital)) OR (((TI hospitalized OR AB hospitalized) OR (TI hospitalised OR AB hospitalised) OR (TI admitted OR AB admitted)) N3 ((TI adult OR AB adult) OR (TI adults OR AB adults) OR (TI patient OR AB patient) OR (TI patients OR AB patients) OR (TI person OR AB person) OR (TI persons OR AB persons) OR (TI people OR AB people))) OR ((TI admitted OR AB admitted) N2 ((TI patient OR AB patient) OR (TI patients OR AB patients))) | 864991 |
| #9  Settings OR'd | S7 OR S8 | 868822 |
| #10  Combining of major concepts | S4 AND S5 AND S6 AND S9 | 13627 |
| #11  *Remove Pediatrics only literature* | S10 NOT (((MH "Adolescence+") OR (MH "Child+")) NOT (MH "Adult+")) | 13627 |
| #12  *Qualitative designs* | (MH "Qualitative Studies+") OR (MH "Focus Groups") OR (MH "Interviews+") OR ((TI qualitative OR AB qualitative) OR (TI qualitatively OR AB qualitatively) OR (TI "focus group" OR AB "focus group") OR (TI "focus groups" OR AB "focus groups") OR (TI "mixed method" OR AB "mixed method") OR (TI "mixed methods" OR AB "mixed methods") OR (TI "thematic analysis" OR AB "thematic analysis") OR (TI "thematic analyses" OR AB "thematic analyses") OR (TI "content analysis" OR AB "content analysis") OR (TI "content analyses" OR AB "content analyses") OR (TI "key informant" OR AB "key informant") OR (TI "key informants" OR AB "key informants") OR (TI fieldwork OR AB fieldwork) OR (TI "field work" OR AB "field work") OR (TI "grounded theory" OR AB "grounded theory") OR (TI phenomenology OR AB phenomenology) OR (TI phenomenological OR AB phenomenological) OR (TI "lived experience" OR AB "lived experience") OR (TI "lived experiences" OR AB "lived experiences") OR (TI ethnograph OR AB ethnograph) OR (TI ethnographic OR AB ethnographic) OR (TI ethnography OR AB ethnography) OR (TI ethnographies OR AB ethnographies) OR (TI autoethnography OR AB autoethnography) OR (TI autoethnographies OR AB autoethnographies) OR (TI autoethnographic OR AB autoethnographic) OR (TI "oral history" OR AB "oral history") OR (TI "oral histories" OR AB "oral histories")) OR (((TI semi-structured OR AB semi-structured) OR (TI semistructured OR AB semistructured) OR (TI unstructured OR AB unstructured) OR (TI informal OR AB informal) OR (TI in-depth OR AB in-depth) OR (TI indepth OR AB indepth) OR (TI "face to face" OR AB "face to face") OR (TI face-to-face OR AB face-to-face) OR (TI structured OR AB structured) OR (TI guide OR AB guide) OR (TI guides OR AB guides)) AND ((TI interview* OR AB interview*) OR (TI discussion* OR AB discussion*) OR (TI questionnaire* OR AB questionnaire*))) | 462764 |
| #13 | S11 AND S12 | 2278 |
| #14 | S13 NOT PT ( Abstract OR Book OR Book Chapter OR Book Review OR Conference Paper OR Dissertation OR Doctoral Dissertation OR Masters Thesis OR Pamphlet OR Pamphlet Chapter OR Poetry ) | 2228 |

**Database: APA PsycINFO (via Ovid)**
Search date: 8/23/2023
*Note: APA PsycInfo 1806 to August Week 2 2023*

| **Search Set** | **Search Strategy** | **Results** |
| --- | --- | --- |
| #1  *Family* | family/ or extended family/ or family members/ or adult offspring/ or biological family/ or cousins/ or grandparents/ or inlaws/ or exp siblings/ or exp spouses/ or significant others/ or friendship/ or (family or families or relatives or spouse or spouses or spousal or partner or partners or husband or husbands or wife or wives or "adult child" or "adult children" or "adult daughter" or "adult daughters" or "adult son" or "adult sons" or sibling or siblings or sister or sisters or stepsister or stepsisters or brother or brothers or stepbrother or stepbrothers or aunt or aunts or uncle or uncles or cousin or cousins or friend or friends or neighbor or neighbors or neighbour or neighbours or "significant other" or "significant others" or "next of kin" or boyfriend or boyfriends or girlfriend or girlfriends).ti,ab. or ((medical or health or healthcare) adj2 (proxy or proxies or surrogate or surrogates or surrogacy)).ti,ab. | 609205 |
| #2 *Informal Caregivers* | exp caregivers/ and (informal or informally or nonprofessional or non-professional or non-clinical or nonclinical or unpaid).ti,ab. | 4012 |
| #3  *Informal Caregivers* | ((informal or informally or nonprofessional or non-professional or non-clinical or nonclinical or unpaid) adj5 ("care giver" or "care givers" or caregiver or caregivers or caregiving or caretaker or caretakers or "care taker" or "care takers" or carer or carers or careworker or careworker or care workers or careworkers or "care worker" or "care workers" or "support person" or "support persons" or "support people")).ti,ab. or ("lay caregiver" or "lay caregivers" or "lay carer" or "lay carers").ti,ab. or ((patient or patients or hospital) adj3 (visitor or visitors or guardian or guardians or companion or companions)).ti,ab. | 5021 |
| #4 | 1 or 2 or 3 | 612119 |
| #5  *Engagement* | participation/ or communication/ or communication barriers/ or interpersonal communication/ or social communication/ or verbal communication/ or (adherence or adherent or attitude or attitudes or activation or collaborate or collaborates or collaborating or collaboration or collaborations or collaborative or collaboratively or communicate or communicated or communicates or communicating or communication or compliance or compliant or contribute or contributed or contributes or contribution or contributions or conversation or conversations or coproduc* or co-produc* or decision or decision-making or decisions or discuss or discussed or discusses or discussing or discussions or empower or empowered or empowerment or empowers or empowering or engage or engaged or engagement or engages or engaging or expectation or expectations or experience or experiences or facilitate or facilitates or facilitated or facilitating or facilitation or facilitations or include or includes or included or including or inclusion or inclusive or integrate or integrated or integrates or integrating or integration or involve or involved or involvement or involves or involving or "joint effort" or "joint efforts" or partnership or partnerships or participate or participates or participating or participation or perceive or perceives or perception or perceptions or presence or planning or role or roles or support or supported or supports or supporting or supportive or synergy or synergistic or synergetic or talk or talked or talking or talks or teamwork or "team work" or visitation or visitations or "working together").ti,ab. or (("professional-family" or "professional family" or "provider-family" or "provider family" or "physician-family" or "provider-family" or "provider family" or "clinician-family" or "clinician family" or "nurse family" or "nurse-family") adj3 (relation or relations or relationships or interaction or interactions or interacting or communication or communications or communicate or communicated or communicating or preference or preferences or attitude or attitudes or behavior or behaviors or behaviour or behaviours)).ti,ab. or ((family or families or patient or patients) adj2 (centered-care or "centered care" or focused-care or "focused care")).ti,ab. | 3805421 |
| #6  *Patient Age / Characteristics* | exp older adulthood/ or exp aging/ or exp geriatrics/ or exp dementia/ or ("older adult" or "older adults" or "frail adult" or "frail adults" or "old person" or "older person" or "old people" or "older people" or "old folk" or "old folks" or "older folk" or "older folks" or elder or elders or elderly or geriatric or geriatrics or geriatrician or geriatricians or gerontology or gerontologist or gerontologists or dementia or dementias or Alzheimer or alzheimers or Alzheimer?s or "lewy body" or "lewy bodies").ti,ab. or ((aged or aging or senior or seniors) adj2 (citizens or adult or adults or patient or patients or inpatient or inpatients or in-patient or in-patients or people or person or man or men or woman or women or population or populations)).ti,ab. | 349137 |
| #7  *Setting: Acute care* | Intensive care/ or hospital environment/ or ("critical care" or "intensive care" or icu or icus or "coronary care unit" or "coronary care units" or ccu or ccus).ti,ab. or ((hospital or hospitals) adj3 ("urgent care" or "emergency care")).ti,ab. or ((hospital or medical or medicine) adj2 (unit or units or ward or wards)).ti,ab. or ((admit or admits or admitted or admitting or admittance or admission or admissions) and ("acute care" or "acute illness" or "acute illnesses" or "acutely ill" or "emergency care" or "emergency service" or "emergency services" or "emergency department" or "emergency departments" or "emergency room" or "emergency rooms")).ti,ab. | 23290 |
| #8  *Setting:*  *hospitalization* | hospitalized patients/ or hospitalization/ or (inpatient or inpatients or in-patient or in-patients or hospitalization or hospitalisation or hospitalizations or hospitalisations or "in-hospital" or inhospital).ti,ab. or ((hospitalized or hospitalised or admitted) adj3 (adult or adults or patient or patients or person or persons or people)).ti,ab. or (admitted adj2 (patient or patients)).ti,ab. | 226939 |
| #9  Settings OR'd | 7 or 8 | 241137 |
| #10  Combining of major concepts | 4 and 5 and 6 and 9 | 2475 |
| #11  *Qualitative designs* | exp Qualitative Methods/ or exp Focus Groups/ or Grounded Theory/ or Interperative Phenomenological Analysis/ or Narrative Analysis/ or Semi-Structured Interview/ or Thematic Analysis/ or (qualitative or qualitatively or "focus group" or "focus groups" or "mixed method" or "mixed methods" or "thematic analysis" or "thematic analyses" or "content analysis" or "content analyses" or "key informant" or "key informants" or fieldwork or "field work" or "grounded theory" or phenomenology or phenomenological or "lived experience" or "lived experiences" or ethnograph or ethnographic or ethnography or ethnographies or autoethnography or autoethnographies or autoethnographic or "oral history" or "oral histories").ti,ab. or ((semi-structured or semistructured or unstructured or informal or in-depth or indepth or "face to face" or face-to-face or structured or guide or guides) and (interview* or discussion* or questionnaire*)).ti,ab. | 460787 |
| #12 | 10 and 11 | 472 |
| #13 | limit 12 to (peer reviewed journal and "0110 peer-reviewed journal") | 412 |
